# Characteristics and scoring method of computed tomography in open-globe injuries

**DOI:** 10.1186/s12886-023-03269-6

**Published:** 2024-01-02

**Authors:** Hongling Chen, Xuemin Jin, Zhongqiang Zhou, Xianliang Zhang, Junjun Han, Ling Wang

**Affiliations:** 1 Department of Ophthalmology, Henan Eye Institute, Henan Eye Hospital, Henan Provincial People’s Hospital, People’s Hospital of Zhengzhou University, Zhengzhou, China; 2 Department of Medical Imaging, Henan Provincial People’s Hospital, People’s Hospital of Zhengzhou University, Zhengzhou, China; 3 Department of Ophthalmology, The First Affiliated Hospital of Zhengzhou University, Zhengzhou, China

**Keywords:** Computed tomography, Open-globe injuries, Score

## Abstract

**Background:**

Open-globe injuries (OGIs) remain the important cause of visual impairment and loss in all ages. Computed Tomography (CT) is a useful and common tool in the evaluation of the injuries of the eyeball. Prognostic value of CT scan in OGIs has been evaluated in many studies. However, there is no published consistent systematic scoring method for CT scan in OGIs. The purpose of this study was to evaluate the CT characteristics of OGIs and build a scoring method according to the CT scans which may aid the clinicians in management of OGIs.

**Methods:**

Retrospective chart review of inpatients with clinical diagnosis of OGIs between 2017 and 2021 at Department of Ophthalmology, Henan Eye Institute, Henan Eye Hospital, Henan provincial People’s Hospital (Zhengzhou, China).

**Results:**

There were 1120 eyes from 1117 patients included in our study. The mean age was 35.7 ± 21.9 years with the range from 1 to 91 years. Significant male predominance was noted (889, 79.6%). CT scans of the OGIs were evaluated. Abnormality of anterior segment, posterior segment, and globe contour and volume were graded respectively. The most serious abnormality of anterior segment, posterior segment, and globe contour and volume were grade 3, 4 and 3 respectively and score 3, 4 and 3 respectively. Score of the CT scans of an open-injured globe ranged from 0 to 10. The correlation coefficient between the score and wound length was 0.798. The correlation coefficient between the score and final visual acuity was 0.799. In 78 eyes with 0 score, 70 eyes (89.7%) gained final visual acuity of 0.3 or better. In 31 eyes with 10 score, 20 eyes (64.5%) underwent evisceration of the eye globe and 10 eyes got visual acuity of no light perception and 1 eye lost to follow-up.

**Conclusions:**

CT scans is a useful tool in evaluating the severity of an open-injured globe. Scoring of the CT scans of an open-injured globe is a meaningful attempt and it may provide useful prognostic information regarding the outcome of an open-injured globe.

## Background

Open-globe injuries (OGIs) refer to full-thickness injuries of the eyewall (cornea and/or the sclera) [1]. OGIs remain the important cause of visual impairment and loss in all ages. Although some cases of OGIs result in significant recovery, severe OGIs can lead to complete loss of ocular architecture and visual potential, requiring evisceration or enucleation of the eye [2–4]. In our previous study, 53 eyes (8.5%) with severe OGIs were eviscerated at primary management, 137 eyes (21.9%) were eviscerated finally after once or more surgeries [3]. So it is meaningful to evaluate the severity of an open-globe injury eye before planning the surgical exploration and communicate with patients. However, comprehensive ophthalmologic assessment for an injured eye is challenging due to periorbital soft-tissue swelling, poor patient cooperation and altered mental status due to concomitant head trauma or the uses of mind-altering mediations. Subjecting patients with globe rupture to an aggressive ophthalmologic examination can even worsen the initial injury [5].

Computed Tomography (CT) is the gold standard for orbital trauma assessment. CT allows the visualization of the ocular globe content and structures [6]. Orbital CT scan could provide valuable information on the integrity of the globe and intraocular structures [7]. Although CT scan is necessary to identify fractures and IOFB, thorough eye examination is of utmost importance.

The purpose of our study was to evaluate the CT characteristics of OGIs patients and tentatively develop a score method according to the CT scans which may aid the clinicians in management of OGIs.

## Methods

### Participant selection and recruitment

We retrospectively chart reviewed all the patients diagnosed and treated at Department of Ophthalmology, Henan Eye Institute, Henan Eye Hospital, Henan provincial People’s Hospital (Zhengzhou, China) between January 1, 2017 and December 31, 2021. Inclusion criteria included clinically diagnosed untreated open-globe injury eyes, eyes with CT scans performed at Henan provincial People’s Hospital. Exclusion criteria included eyes with intraocular foreign body (IOFB), eyes had been treated at other hospital, eyes without CT scans or eyes with CT scans performed at other hospital.

Initial ophthalmology consultation notes, hospital records, details of the primary, subsequent surgical interventions, and outpatient follow-up records were reviewed. Demographics, including age and gender, wound characteristics (i.e., mechanism, causes, wound size, and locations), and visual acuity (VA) (presenting and final VA) were collected. The final VA was defined as the VA at the end of the follow-up (over 6 months).

### CT scoring

CT scans were evaluated by one experienced radiologist (Wang L) and one experienced ophthalmologist (Chen H). CT scoring standard was based upon 3 aspects of the eyeball: anterior segment (A), posterior segment (P), and globe contour and volume (G) (Table 1).

**Table 1:** Scoring standard for CT scans according to radiological features

Score	Anterior Segment (A)	Posterior Segment (P)	Globe Contour and Volume (G)
0	Negative finding on CT scans	Negative finding on CT scans	Negative finding on CT scans
1	Subtle changes such as alteration or asymmetry in the AC depth, abnormal density within the AC, hypoattenuating lens.	Subtle changes such as air bubbles, spot or sheet increased attenuation less than 1/3 of the posterior segment.	Subtle globe contour irregularity, globe volume shrunk to 4/5 or more of normal.
2	Moderate structural disorder such as air bubbles, obvious alteration or asymmetry in the AC depth, abnormal density within the AC, and lens subluxation.	Moderate changes such as air bubbles, increased attenuation with 1/3 to 4/5 of the posterior segment.	Moderate globe contour irregularity, globe volume shrunk to 1/3 to 4/5 of normal.
3	Severe structural disorder such as flat AC, contour deformity, lens be absent or displaced.	Homogenous attenuation with 4/5 or more of the posterior segment.	Globe contour be deformed, globe volume shrunk to 1/3 or less of normal.
4		Structures in mess, heterogenous dense attenuation with the whole posterior segment.	

Normal CT imaging was recorded as A0P0G0 and score 0.

**Fig. 1:**
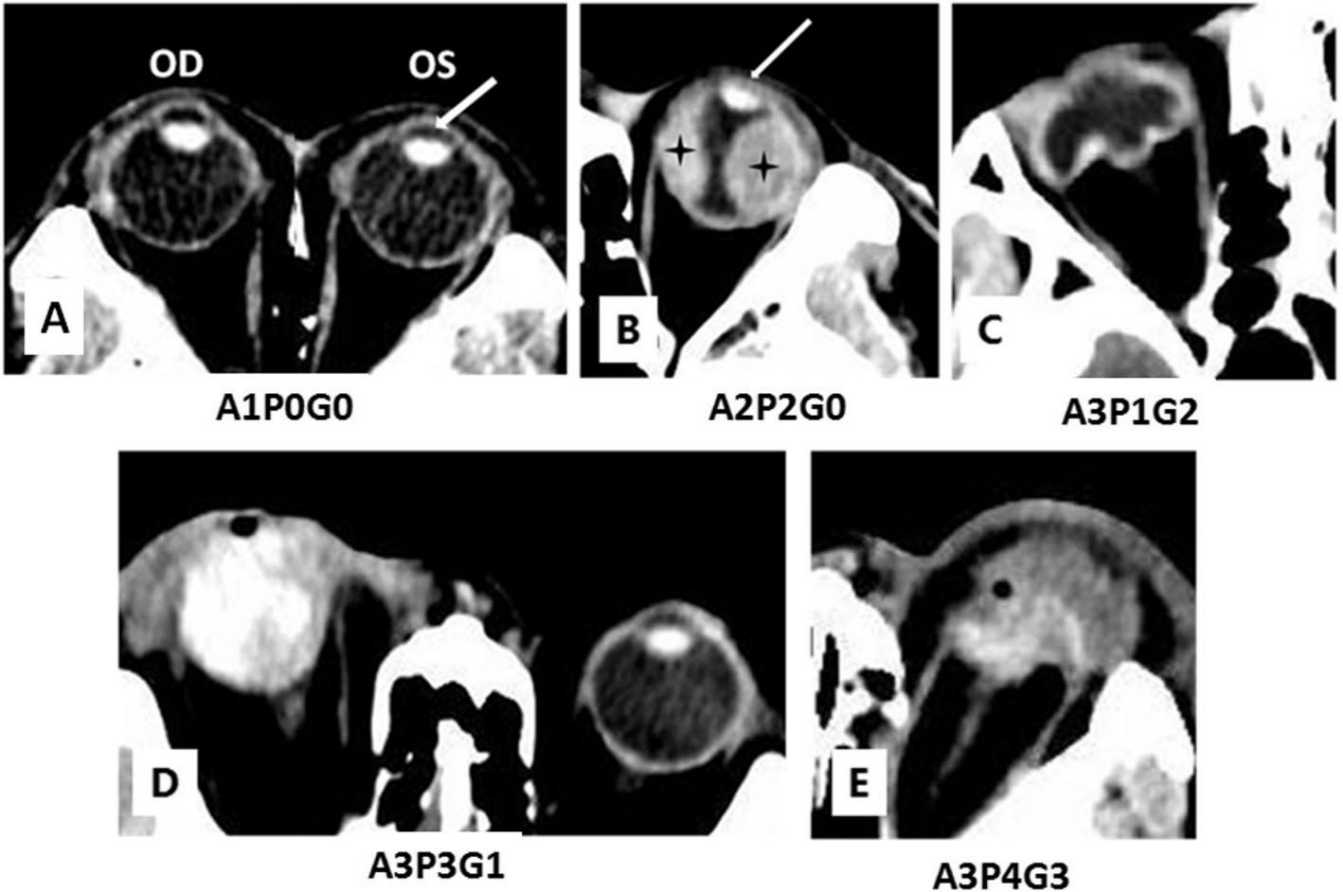
**A** Left open-globe injury after being hit in the left eye with a sheet metal. It was a 6 mm area 1 wound. Axial CT scan of the eyes shows relative shallow anterior chamber depth (arrow) of the left globe compared to that of the right eye. **B** Left open-globe injury after being hit in the left eye with metal block. It found to be a 20 mm wound involving area 1 and 2 during suture surgery. Axial CT scan of the eyes shows relative shallow anterior chamber depth of the left globe compared to that of the right eye. The patient was lost follow-up. **C** Right open-globe injury after hitting on the door. It was a 12 mm area 1 wound. Axial CT scan of the eyes shows the absence of the lens. It was found that both lens and iris were absent during suture surgery. Volume of the globe reduced to about half of normal. Fortunately, there was not massive hemarrhage in the vitreous chamber. After suture surgery, the final visual acuity achieved 0.1 and no further surgery was carried out during over 3 years of follow-up. **D** Right open-globe injury after being hit in the right eye by an exploding glass bottle. Axial CT scan of the eyes shows the globe is full of massive hemarrhage. It found to be a 25 mm wound involving area 1, 2 and 3 during suture surgery. After suture surgery, the injured eye became phthisical eventually during follow-up. **E** Left open-globe injury after being hit by exploding fireworks. It found to be a 35 mm wound involving area 1, 2 and 3 during suture surgery. Forty five days later, the injured eye was eviscerated

CT imaging with subtle changes of anterior segment (such as alteration or asymmetry in the anterior chamber (AC) depth, abnormal density within the AC, hypoattenuating lens), normal posterior segment and normal globe contour and volume was recorded as A1P0G0 (Fig. 1 A) and score 1.

CT imaging with moderate structural disorder of anterior segment (such as air bubbles, obvious alteration or asymmetry in the AC depth, abnormal density within the AC, and lens subluxation), moderate changes (such as air bubbles, increased attenuation with 1/3 to 4/5 of the posterior segment) of posterior segment and normal globe contour and volume was recorded as A2P2G0 (Fig. 1 B) and score 4.

CT imaging with severe structural disorder of anterior segment (such as flat AC, contour deformity, lens be absent or displaced), subtle changes of the posterior segment (such as air bubbles, spot or sheet increased attenuation less than 1/3 of the posterior segment) and moderate changes of the globe contour and volume (moderated changes of globe contour irregularity, globe volume shrunk to 1/3 to 4/5 of normal) was recorded as A3P1G2 (Fig. 1 C) and score 6.

CT imaging with severe structural disorder of anterior segment, homogenous attenuation with 4/5 or more of the posterior segment and subtle globe contour irregularity, globe volume shrunk to 4/5 or more of normal was recorded as A3P3G1 (Fig. 1 D) and score 7.

CT imaging of the most severe injury was recorded as A3P4G3 (Fig. 1 E) and score 10.

### Visual acuity (VA) score

Visual acuity was documented according to standard visual acuity chart (decimals). In order to make statistics convenient, we converted VA to score 0 to 10 (Table 2). For example, VA of no light perception (NLP) score 0, light perception (LP) score 1, hand motion (HM) score 2, counting finger (CF) score 3, and so on. VA of 1.0 or better score 10.

**Table 2:** VA scoring, distribution of initial and final VA in this study

Score	VA	Initial		Final	
		n	%	n	%
0	NLP	307	27.4	221	19.7
1	LP	245	21.9	28	2.5
2	HM	204	18.2	82	7.3
3	CF	104	9.3	70	6.3
4	(CF-0.1)	53	4.7	63	5.6
5	[0.1–0.2]	60	5.4	129	11.5
6	[0.25–0.3]	20	1.8	47	4.2
7	[0.4–0.5]	27	2.4	72	6.4
8	[0.6–0.7]	3	0.3	66	5.9
9	[0.8–0.9]	4	0.4	70	6.3
10	≥1.0	4	0.4	101	9
	unable to test VA	89	8	28	2.5
	Lost to follow-up			143	12.8
Total		1120	100	1120	100

### Statistical analysis

Statistical analysis was performed with SPSS v.19.0 software (SPSS, Inc., Chicago, IL, USA).

Ethics approval for the study was granted by Henan Eye Institute, Henan Eye Hospital, Henan provincial People’s Hospital Human Research Ethics Committee. Ethics approval number is HNEEC-2023 (09). The study adhered to the tenets of the Declaration of Helsinki.

## Results

A total number of 1117 patients (1120 eyes) were included in the study. Significant male predominance was noted (889, 79.6%). Among unilateral injuries, no significant difference was observed between the affected eyes (right eye 50.5% vs. left eye 49.2%), 0.3% of all cases were bilateral. The mean age was 35.7 ± 21.9 years with the range from 1 to 91 years. Of the 1120 eyes, 671 (59.9%) were diagnosed with penetration, 447 (39.9%) were rupture, and 2 eyes (0.2%) were perforation. Nail/wire accounted for 14.7% followed by Wood/branch/bamboo (10.3%), traffic accident (9.3%) and fall/tumble (9.0%) of all the OGIs (Table 3).

**Table 3:** Causes of open globe injuries

Cause	n	%
Nail, wire	164	14.7
Wood, branch, bamboo	115	10.3
Traffic accident	104	9.3
Fall, tumble	100	9.0
Scissors, knife	84	7.5
Metal fragments, Metal block, sheet metal	62	5.6
Metal bar/tube	49	4.4
Fireworks, firecrackers	45	4.0
Emery cutter, grinding wheel, electric saw	45	4.0
Stationery	44	3.9
Glass	42	3.8
Violence	29	2.6
Toys	28	2.5
Flying stone	25	2.2
Lighter, bottle, bulb explosion	12	1.1
Plastic pipe/block/sheet	11	1.0
Tool	8	0.7
Cup	6	0.5
Finger, nail	5	0.5
Elbow	5	0.5
Bird, cock	5	0.5
Spring	3	0.3
Toy bullet	2	0.2
Battery explosion	2	0.2
Goats attack	2	0.2
Straw	2	0.2
High pressure pipe	2	0.2
Tyre explosion	1	0.1
Cellphone	1	0.1
Crab	1	0.1
Knee	1	0.1
Remote control	1	0.1
Water pump explosion	1	0.1
Others and unknown	110	9.8
Total	1117	100

Of all the OGIs, 61 eyes developed post-traumatic endophthalmitis and 3 eyes sympathetic ophthalmia.

The correlation coefficient between CT score and wound size was 0.798. Mean wound size of 0 and 10 CT score groups was 4.95 ± 2.04 mm and 27.71 ± 7.99 mm respectively (Table 4).

**Table 4:** Wounds size, managements, and outcomes with different CT scores

CT score	eyes	wound size (mm)	Evisceration		Endophthalmitis		Eyes underwent vitrectomy		Penetration/ Perforation		Rupture		Mean final VA^a^		VA of NLP^a^		≥0.3^a^	
			n	%	n	%	n^c^	%	n	%	n	%	n	VA Score	n	%	n	%
0	111	4.95 ± 2.04	0	0	5	4.5	5(3)	4.5	106	95.5	5	4.5	78	8.55 ± 1.78	0	0	70	89.7
1	303	5.81 ± 3.19	2^b^	0.7	37	12.2	49(26)	16.2	286	94.4	17	5.6	214	7.34 ± 2.27	1	0.5	155	72.4
2	170	7.95 ± 3.90	0	0	13	7.7	51(12)	30.0	132	77.7	38	22.3	135	5.87 ± 2.48	3	2.2	71	52.6
3	103	10.45 ± 4.02	3	2.9	3	2.9	35(3)	34.0	69	67	34	33	77	4.64 ± 2.37	5	6.5	19	24.7
4	80	13.48 ± 5.19	1	1.3	2	2.5	52(2)	65.0	33	41.3	47	58.7	68	3.90 ± 2.07	2	2.9	11	16.2
5	87	15.94 ± 5.80	6	6.9	1	1.2	46(1)	52.9	17	19.5	70	80.5	79	2.38 ± 2.16	25	31.7	7	8.9
6	92	18.87 ± 8.42	21	22.8	0	0	43	46.7	12	13	80	87	79	1.16 ± 1.64	45	57	0	0
7	70	20.83 ± 6.91	17	24.3	0	0	34	48.6	10	14.3	60	85.7	65	0.60 ± 1.16	46	70.8	1	1.5
8	34	23.77 ± 7.72	14	41.2	0	0	12	35.3	3	8.8	31	91.2	32	0.25 ± 0.67	28	87.5	0	0
9	39	25.28 ± 7.14	16	41.0	0	0	15	38.5	3	7.7	36	92.3	37	0.35 ± 0.75	30	81.1	0	0
10	31	27.71 ± 7.99	20	64.5	0	0	5	16.1	3	9.7	28	90.3	30	0.10 ± 0.40	28	93.3	0	0
total	1120		100		61		347(47)		674		446		894		213		334	

^a^Except endophthalmitis, unable to test VA, and Lost to follow-up, ^b^Both eyes were endophthalmitis, ^c^ The number in brackets indicated eyes with endophthalmitis

Endophthalmitis happened most frequently (12.2%) in 1 CT score group with the wound size of 5.81 ± 3.19 mm.

The correlation coefficient between CT score and final VA (except eyes with endophthalmitis) was 0.799. The mean final VA score of 0 CT score eye was 8.55 ± 1.78 (0.6–0.9), and the mean final VA score of 10 CT score eye was 0.10 ± 0.40 (NLP-LP). In 78 eyes (except endophthalmitis, unable to test VA, and Lost to follow-up) with 0 CT score, 70 eyes (89.7%) gained final visual acuity of 0.3 or better, and in 31 eyes with 10 CT score, 20 eyes (64.5%) underwent evisceration of the eye globe and 8 eyes got visual acuity of NLP, 2 eyes unable to test VA and 1 eye lost to follow-up (Table 4).

## Discussion

Orbital CT features of OGIs have been reported previously in many studies [8–13]. Its diagnostic values have been study in previous studies too [8, 14–17]. Some studies found that CT is not sensitive enough to be solely relied upon for diagnosis of all open globe injuries [8, 14]. CT findings only complement clinical findings, increasing the clinician’s overall ability to make an accurate diagnosis [8]. Pikkel et al. [18] described three patients with severe ocular trauma resulting in ocular perforation, in whom CT, performed prior to thorough ocular examination, showed no sign of perforation. In the present study, 9.9% eyes score 0 which mean negative finding on CT scans. So there is no replacement for a thorough clinical examination mainly in trauma cases.

Apart from its limitations in diagnosis of some open-globe injuries, CT is a useful tool in the evaluation of the injuries of the eyeball. Prognostic value of CT scan in OGIs has been evaluated in many studies [5, 19, 20]. CT imaging is invaluable to be able to make a relatively confident prediction of clinical findings and decide upon the necessity for acute ophthalmic surgical intervention [20]. With the help of CT and preoperative clinical data, radiologist can predict visual acuity after open globe injury [5].

In fact, both radiologist and ophthalmologist should be prepared to rapidly recognize the severity of the injuries according to the CT imaging. Building a score system of the CT imaging that can briefly demonstrate the characteristic and severity of the injury is very necessary.

However, we did not find similar score system in published literatures. So we evaluated the orbital CT scans of 1120 eyes with OGIs, and tentatively built a score system of the CT imaging regard to the abnormalities of the contents and integrity of the globe. This score system include three aspects s of the eyeball: anterior segment (A), posterior segment (P), and globe contour and volume (G) (Table 1). For example, normal CT imaging was recorded as A0P0G0 and score 0; CT imaging of the most severe injury was recorded as A3P4G3 and score 10.

CT imaging with 0 score usually means better results (89.7% eyes gained VA OF 0.3 or better) and CT imaging with 10 score usually means very bad results (64.5% eyes got eviscerated and almost all the rest eyes got VA of NLP during follow-up).

In fact, for 10 CT score eyes, the best management of the first surgery was evisceration. However, most patients in our hospital strongly disagreed to perform eye removal surgery at first management.

Low CT score means small wound size and high possibility of penetration or perforation with the outside-in mechanism which increased the possibility of endophthalmitis. CT imaging with 0 score usually means self-sealing wounds and relatively low risk of endophthalmitis (4.5%). On the other side, CT imaging with 1 score usually means open wounds and highest risk of endophthalmitis (12.2%).

The most important characteristic of this score system (APG) is that it can briefly and clearly manifest the involved part (anterior segment, posterior segment), globe contour and volume of the eyeball. The involvement of the posterior segment is bad prognosis predictors. So this APG CT score system can helps clinicians to make a rational choice of surgical interventions and makes the communication between ophthalmologists, ophthalmologist and patient, ophthalmologist and radiologist very convenient.

This APG CT score system is far from perfect. One of the defects of this CT score system is that the CT score was evaluated by human (radiologist and ophthalmologist), so bias was unavoidable. Recent study of Liu et al. brings inspiration for us, in which an active shape model (ASM) segmentation (vitreous cavity, lens, sclera, AC, and cornea) [9]. Intelligent quantitative CT score method deserves further study.

Another limitation of this study is inherent to the retrospective nature of the study design. Additionally, we excluded patients with IOFB, which their enrollment would have increased the uncertainty of the results of the globe. This study was the first attempt about scoring of CT in OGIs. IOFB is an ophthalmic true emergency, needing immediate surgical treatment. However, even with immediate appropriate management, IOFB complications can lead to severe visual impairment or blindness. In the future study, we would like to analyze the characteristics and scoring of CT in IOFB independently.

In proposing a scoring method, it should be as simple as possible. This study might be a basis for such scoring method. In the future, we would devote to simplify it and carry out prospective studies on the intelligent quantitative CT score system.

## Conclusions

CT scans is a useful tool in evaluating the severity of an open-injured globe. Scoring of the CT scans of an open-injured globe is a meaningful attempt and it may provide useful prognostic information regarding the outcome of an open-injured globe. It is worth our further study.

## Data Availability

The datasets used and analyzed during the current study are available from corresponding author upon reasonable request.

## Abbreviations

CT: Computed tomography
OGIs: Open-globe injuries
VA: Visual acuity
IOFB: Intraocular foreign body
AC: Anterior chamber
NLP: No light perception
LP: Light perception
HM: Hand motion
CF: Counting finger.
